# Artificial intelligence for strengthening healthcare systems in low- and middle-income countries: a systematic scoping review

**DOI:** 10.1038/s41746-022-00700-y

**Published:** 2022-10-28

**Authors:** Tadeusz Ciecierski-Holmes, Ritvij Singh, Miriam Axt, Stephan Brenner, Sandra Barteit

**Affiliations:** 1 Heidelberg Institute of Global Health (HIGH), Faculty of Medicine and University Hospital, Heidelberg University, Heidelberg, Germany; 2 University of Cambridge, School of Clinical Medicine, Addenbrooke’s Hospital, Cambridge, CB2 0SP UK; 3 Imperial College London, Faculty of Medicine, Sir Alexander Fleming Building, London, SW7 2DD UK

**Keywords:** Health policy, Translational research

## Abstract

In low- and middle-income countries (LMICs), AI has been promoted as a potential means of strengthening healthcare systems by a growing number of publications. We aimed to evaluate the scope and nature of AI technologies in the specific context of LMICs. In this systematic scoping review, we used a broad variety of AI and healthcare search terms. Our literature search included records published between 1st January 2009 and 30th September 2021 from the Scopus, EMBASE, MEDLINE, Global Health and APA PsycInfo databases, and grey literature from a Google Scholar search. We included studies that reported a quantitative and/or qualitative evaluation of a real-world application of AI in an LMIC health context. A total of 10 references evaluating the application of AI in an LMIC were included. Applications varied widely, including: clinical decision support systems, treatment planning and triage assistants and health chatbots. Only half of the papers reported which algorithms and datasets were used in order to train the AI. A number of challenges of using AI tools were reported, including issues with reliability, mixed impacts on workflows, poor user friendliness and lack of adeptness with local contexts. Many barriers exists that prevent the successful development and adoption of well-performing, context-specific AI tools, such as limited data availability, trust and evidence of cost-effectiveness in LMICs. Additional evaluations of the use of AI in healthcare in LMICs are needed in order to identify their effectiveness and reliability in real-world settings and to generate understanding for best practices for future implementations.

## Introduction

Rapid technological developments of the past few decades have paved the way for an abundance of technologies that have and continue to revolutionise medicine and healthcare^1–3^. The field of artificial intelligence (AI), in particular, benefits largely from the expanding accessibility of the internet, the progression in software system development, and the fast advancement of microprocessor technology that translated into a variety of widely available devices including tablets, smartphones, laptops and virtual reality appliances^4^. With a widely recognised and accepted definition still underway^5^, this paper uses the definition by Russel and Norvig which describes AI as the wider field of “designing and building intelligent agents that receive precepts from the environment and take actions that affect that environment”^6^.

Particularly relevant AI technologies in medicine and healthcare include knowledge engineering, machine learning (e.g. precision medicine, neural network models), natural language processing, rule-based expert systems, surgical robots, or image and signal processing^7^. Medical education, clinical practice and healthcare delivery have all benefited from these technology advancements, which have offered new techniques and methodological approaches. AI is revolutionising the foundations of healthcare with its potential to improve both the scope and accessibility of healthcare provision at a global scale^8,9^.

Given these technological developments, AI has the potential to substantially change the role of how medical care and public health programmes might be implemented in the near future, especially in health systems where the distributions of and access to care have so far been challenging^3,10^. In low- and middle-income countries (LMICs), the value of AI is seen in its potential to build health systems by supporting and standardising clinical judgement and applying healthcare processes more objectively with a data-oriented approach^11^. Furthermore, given the shortages of skilled health workers in areas such as sub-Saharan Africa, where medical education capacities are limited^12^, AI-powered clinical tools could represent one way to increase quantity and quality of medical care^13^. However, current AI applications and machine learning still require large amounts of complete and regularly updated datasets, which still remain scarce for most LMICs^14^. While reports on the application of different AI technologies in LMICs continue to grow, the actual evidence base has so far not been reviewed. The scope and extent of implemented AI remains unclear, or whether AI technologies have proven to have potential for healthcare delivery in LMICs.

The goal of this systematic scoping review is therefore to review and map existing literature on health-specific AI applications and to summarise currently available evidence of AI’s efficacy across LMICs. To allow for a comprehensive outline of AI technologies applied to both medical practice and healthcare delivery, this paper systematically reviews and identifies all relevant literature across a wide range of AI applications, health systems, and LMICs. A further focus is on strengths, weaknesses and perceptions of the application of AI in healthcare in LMICs, exploring the following questions:

- What are the effects of current AI-based technology on healthcare provision (e.g. diagnosis, treatment, health outcomes, provider or patient time, costs, etc.)?
- What are the experiences of providers and patients with respect to the application of current AI-based healthcare technology (e.g. acceptance, perceived usefulness, trust in technology, feasibility to implement and integrate, etc.)?
- What are key elements that support or challenge AI implementation in the LMIC healthcare context?

## Results

### Eligible records

**Fig. 1:**
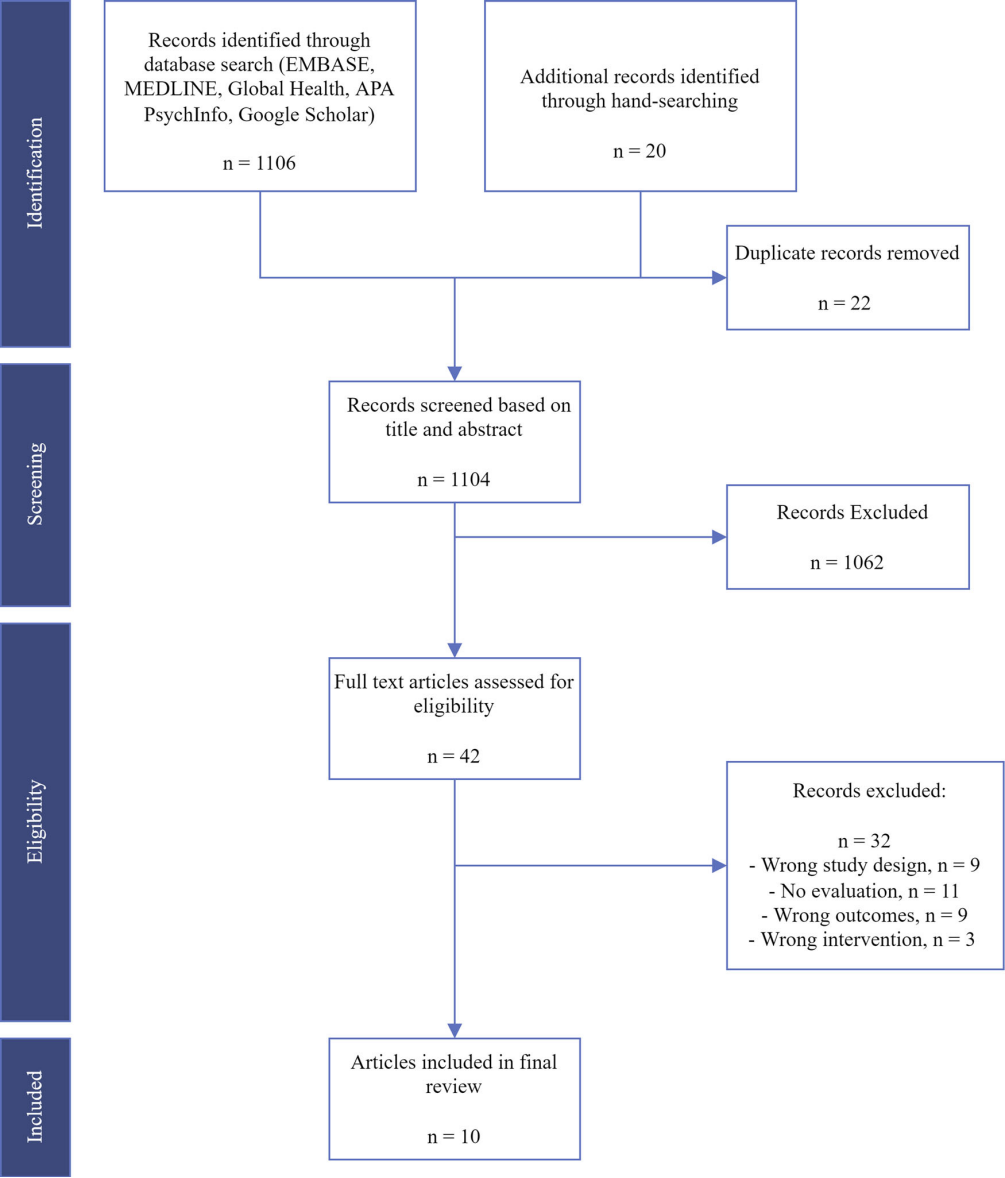
PRISMA Flowchart of studies included in the systematic review. Flowchart of study identification, exclusion based on titles and abstracts, and inclusion in the final review after assessing full texts.

Our database and handsearch identified a total of 1126 articles, of which 1104 were included in title and abstract review after removal of duplicates (see Fig. 1 for details). The final sample of peer-reviewed articles entering analysis included a total of ten studies, described in Table 1. A list of references for the included studies is available in Supplementary Note 2.

**Table 1:** Research contexts of studies that evaluated Artificial Intelligence implementations in low- and middle-income countries.

Study	Study focus	Study design	Study outcome	Study methodology	Analytical approach	Study sample
Love et al.^17^	Training of non-radiologist healthcare workers in a Mexican hospital to use AI CADx system to triage palpable breast lumps for further examination by oncologists	Cross-sectional study	The concordance between AI’s scoring of breast lumps, when used by non-radiologist health workers, and a radiologist’s BI-RADS of the breast lumps	Quantitative	Predictive analysis	32 palpable breast lumps, examined by three non-radiologist health workers using AI tool
Zhou et al.^20^	The performance of IBM Watson for Oncology when producing treatment plans for cervical cancer patients in a Chinese university hospital	Cross-sectional study	Concordance between AI recommended and ‘for consideration’ treatments, and treatments implemented by physicians	Quantitative	Inferential analysis	362 retrospective cancer patients; single case-study patient
Kisling et al.^21^	The ability of MD Anderson’s ‘Radiation Planning Assistant’ to automate the production of safe and effective radiotherapy treatment plans for cervical cancer patients in two South African hospitals	Cross-sectional study	The acceptability and accuracy of AI generated treatment plans by specialists in gynaecologic radiation oncology, in addition to AI run-time for generating plans	Quantitative	Descriptive analysis	14 cervical cancer patients
Ugarte-Gil et al.^15^	The utility of the ‘eRx’ CADx system for TB diagnostics in primary care clinics in Peru, and the challenges of implementing such a system from the user’s perspective	Cross-sectional study	The experiences of nurses and doctors with the technology, including barriers and complications that arose	Mixed methods	Descriptive and content analysis of reported provider experiences	Seven nurses and five doctors working at primary care hospital or health centres
Garzon-Chavez et al.^18^	The implementation of an AI-assisted CT screening tool for COVID-19 patient triage in the workflows of an Ecuadorian hospital	Cross-sectional study	The sensitivity and specificity of AI-assisted CT screening to correctly identify likely COVID-19 positives as confirmed by RT-PCR test	Quantitative	Predictive analysis	75 chest CTs for patients with laboratory confirmed SARS-CoV-2 diagnosis
Fan et al.^23^	The real-world use of an AI health chatbot for primary care self-diagnosis in China, including issues and barriers in their usage, and user experiences	Cross-sectional study	The characteristics of users, length and frequency of chatbot sessions, health concerns presented, and user feedback	Mixed methods	Descriptive and inferential analysis of chatbot sessions; content analysis of user feedback	47,684 consultation sessions initiated by 16,519 users
Ganju et al.^16^	The use of an Indian child health and nutrition education mHealth app’s usage data to predict user churn and target interventions to improve user engagement	Cross-sectional study	The engagement and retention of users with mHealth app	Quantitative	Predictive analysis of user churn	45,000 mHealth app users
Wang et al.^22^	The experiences of physicians using a clinical decision support system for diagnostic assistance and treatment suggestions in rural primary care clinics in China	Cross-sectional study	The perspectives of physician using the AI tool, including the perceived challenges, limitations, trustworthiness, and usefulness of the tool	Qualitative	Content analysis	22 clinicians from rural primary care clinics
Wang et al.^24^	The anonymous implementation of a social support chatbot for online pregnancy healthcare in a community in China	Cross-sectional study	The AI’s response rate and response time to community members, and emotional valence of responses, compared with responses from other community members	Mixed methods	Descriptive analysis, content analysis	3445 users of YouBaoBao online pregnancy healthcare community
MacPherson et al.^19^	The improvement in patient outcomes and cost-effectiveness of implementing an X-ray CADx screening tool for TB in a primary clinic in Malawi, as part of an existing HIV-TB screening programme	Randomised controlled trial	The time in days, up to 56 days, to TB treatment initiation compared with standard-of-care and HIV treatment arm, and ICER of TB screening treatment arm	Quantitative	Economic analysis	1462 resident adults attending health centre reporting TB symptoms with no history of TB

### Study characteristics

Four studies were conducted in China, while the other six represent a range of LMICs across Latin America, South Asia and Sub-Saharan Africa (see Table 2 for a summary of key characteristics). Overall, a majority of studies (*n* = 8, 80%) were conducted in the context of upper-middle-income countries. All identified studies have been published since 2018 onwards. While most studies are based on cross-sectional designs, these varied in their quantitative and qualitative methodologies. Study populations ranged from 12 in a clinical research setting to 45,000 in research involving mHealth platforms^15,16^.

**Table 2:** Key characteristics of included studies.

Characteristics	No. of studies (%)
Country	
China	4 (40)
Ecuador	1 (10)
India	1 (10)
Malawi	1 (10)
Mexico	1 (10)
Peru	1 (10)
South Africa	1 (10)
Country income groups	
Low-income country	1 (10)
Lower-middle-income country	1 (10)
Upper-middle-income country	8 (80)
Study year (last year of study period)	
2020	2 (20)
2019	6 (60)
2017	1 (10)
2016	1 (10)
Study design	
Cross-sectional	9 (90)
Randomised control trial	1 (10)
Study methodology	
Qualitative	1 (10)
Quantitative	6 (60)
Mixed-Methods	3 (30)
Studied sample sizes	
Small (<100 subjects)	4 (40)
Medium (between 100–500 subjects)	2 (20)
Large (>500 subjects)	4 (40)

### Features of studied AI technologies

Table 3 summarises the features of the studied AI technologies. Of the AI technologies studied in the reviewed articles, three were applied to the care of communicable diseases (two to HIV/tuberculosis, one to COVID-19), four to the care of non-communicable illnesses (three to various cancers, one to child nutrition), and three to general primary healthcare including pregnancy care. Within their clinical context, three technologies were applied to patient triage, four to screening or diagnostics, and three to care planning or provision. Of these, three tools assisted with triage and screening tasks performed by frontline health workers^17–19^. Four tools assisted physicians with diagnoses, clinical decision making and treatment planning^15,20–22^. Two articles studied the use of chatbots by individuals in the community, one being an ‘AI Doctor’ for primary care self-diagnosis^23^, and another offering social support messages on a health forum^24^. Two articles examined AI technologies used in distributing health educational information and support on child nutrition or pregnancy-related care with target populations in the community^16,24^.

**Table 3:** Key features of implemented AI technologies.

AI features	No. of studies (%)
Disease-specific applications	
Communicable diseases	3 (30)
Non-communicable diseases	4 (40)
Either	3 (30)
Clinical applications	
Patient triage	2 (20)
Diagnosis or screening	4 (40)
Care planning	2 (20)
Care provision	1 (10)
Transparency of AI approaches used in studies	
Algorithms	5 (50)
Training data	5 (50)
Interpretability of AI algorithms/approaches used	
Black box	7 (70)
Interpretable output	3 (30)
Use category of Artificial Intelligence Implementation	
Frontline Health Worker Virtual Assistant	3 (30)
Physician Clinical Decision Support	4 (40)
Patient Virtual Health Assistant	3 (30)
Groups interacting with AI	
Individuals/patients	3 (30)
Non-physician healthcare workers	3 (30)
Physicians	5 (50)

#### Transparency of data and algorithms used in training AI tools

Overall, included studies varied regarding the extent to which datasets and algorithms used in the training and testing of AI tools were made transparent. Further, none of the datasets described in any of these studies were immediately accessible to the public in full. Five studies, however, provide reference to the datasets used^15–18,24^, and five studies described the AI algorithms used in detail^15–17,21,24^. Studies using commercially available products provided limited or no information on their respective datasets and algorithms^18–20,22,23^. Information gathered about the datasets and algorithms used can be found in Supplementary Table 2 and the Supplementary Discussion.

#### Interpretability of AI models

Most AI tools (*n* = 7, 70%) lacked any interpretability of their outputs, using ‘black-box’ algorithms^15–17,21–24^. A total of two AI tools for diagnosing TB or COVID-19 using chest X-rays provided interpretable heatmaps/areas-of-interest on a chest X-ray^18,19^. One study used IBM Watson for Oncology, a cancer treatment planning assistant, which provides relevant literature, such as clinical trial data, for a particular treatment it has recommended - though it is still largely a black-box tool^20,25^.

### Strengths, weaknesses and perceptions of implemented AIs

In the next sections, we focus specifically on cost-savings and improvements in health outcomes, effect on workflows and time to treatment and diagnosis, local adequacy of AI, and user-friendliness, reliability and trust in AI technologies summarised in Table 4.

**Table 4:** Reported strengths and weaknesses of AI tools.

		Dimensions of strength or weakness as reported for studied AI technologies								
Study	AI Tool	High Concordance with Physicians/ Diagnostic Test	Improved Workflows	Improved Diagnostic and Treatment Times	User-friendliness of AI tool	Local Context Accounted For	User Trust in AI Technology	Cost-Effectiveness	Compatibility with Existing Infrastructure	Improved Individual Health Outcomes
Love et al. (2018)^17^	Ultrasound breast lump triage	✓	✓	–	✓	–	–	–	–	–
Zhou et al. (2019)^20^	Cancer treatment planning assistant	o	–	–	–	x	–	–	–	–
Kisling et al. (2019)^21^	Automated radiation planning assistant	✓	✓	–	–	–	–	–	–	–
Ugarte-Gil et al. (2020)^15^	TB chest X-ray computer aided diagnostics	–	x	–	x	x	–	–	x	–
Garzon-Chavez et al (2021)^18^	COVID-19 chest CT computer aided diagnostics	x	✓	–	–	–	–	–	✓	–
Fan et al. (2021)^23^	Chatbot doctor	–	–	–	o	–	o	–	–	–
Ganju et al. (2021)^16^	User churn prediction	–	–	–	–	–	–	–	✓	–
Wang et al. (2021a)^22^	Clinical decision support system	o	o	x	x	x	x	–	x	–
Wang et al. (2021b)^24^	Social support chatbot	–	–	✓	–	–	–	–	–	✓
MacPherson et. al (2021)^19^	TB chest X-ray computer aided diagnostics	✓	–	✓	–	–	–	x	–	✓

Cells marked with an ‘✓’ indicate that the cited article reported this dimension as a strength; cells marked with a ‘x’ indicate that the cited article reported this dimension as a weakness; cells marked with a ‘o’ indicate that the cited article reported a mix of strengths and weaknesses for that particular dimension; cells marked with a ‘-’ indicate insufficient information about a given dimension in the cited article or that this particular dimension is not applicable.

#### Reliability of AI tools

Concordance between the AI tools and physicians was reported in four studies^17,20–22^. Perfect concordance was reported in small samples of triaged breast lumps using ultrasound and radiation treatment plans^17,21^, but also some discordance between clinical decision support systems and the local treatment options available^20,22^.

Concordance of the IBM Watson for Oncology between physicians’ clinical decisions and treatment suggestions varied from 12% to 96% across several cancers^20^. This included cases where a suggested treatment was too expensive, not available, considered to be too aggressive or inconvenient for the patient, or locally available alternatives would have been preferred. Baidu Inc’s ‘Brilliant Doctor’ clinical decision support system made generally good suggestions, but sometimes disagreed with physicians on their first choice of diagnosis and treatment^22^. Participating physicians reported that inadequate care recommendations were usually a result of the system’s poor interoperability with other IT systems, use of inaccurate information, and missing information on patient’s income and insurance background^22^. The misalignment with the local clinical context was attributed to the training protocols used. For example, both tools were trained on data outside of their applied contexts, and thus did not fully account for the local disease incidence and treatment options available^20,22^.

#### Effect on workflows and time to treatment and diagnosis

AI technologies improved workflows in a number of ways. Non-sonographer healthcare workers (HCWs) could be trained in AI-based ultrasound triage, thus reducing the workloads of formally trained sonographers^17^. Similarly, automated radiation treatment planning reduced the time spent by radiation oncologists in making treatment plans^21^. COVID-19 triage was also more time-efficient in an Ecuadorian hospital once an AI computed tomography (CT)-screening tool was implemented^18^. The ‘Brilliant Doctor’ clinical decision support system also had a partially positive impact in rural Chinese primary-care clinics by suggesting diagnostic alternatives to physicians, thus facilitating medical information search and potentially reducing the likelihood of medical errors^22^. Notably, however, higher workloads were reported in clinical settings with low capacity for adopting new AI tools^15,22^.

Shortened time to diagnosis or treatment were reported in two studies. Delft’s ‘CAD4TB’ TB screening tool reduced time to the initiation of treatment compared to standard sputum screening tests in a Malawian primary-care clinic, while a social support chatbot improved response times for individuals seeking social support in online forums in China^19,24^.

#### User-friendliness and compatibility with existing infrastructure

User-friendliness and compatibility with existing infrastructure are quintessential in this context, as healthcare personnel or patients may not be trained or used to using new technologies while being short on time, resources and making potentially life-changing decisions under pressure. These aspects were noted in four of our included studies.

The ‘Brilliant Doctor’ clinical decision support system was found to require too much information from physicians, which was perceived as too time-consuming in a majority of cases^22^. Lacking integration with existing IT systems also resulted in critical laboratory information not being factored into the AI’s decision making process^22^. Physicians in Peruvian TB clinics also reported problems with an app-based TB-diagnostics tool utilising chest X-rays, including issues such as crashes of the app or mistranslations^15^. Poor internet connectivity inside the clinics and the overall limited availability of X-ray viewers throughout clinics impeded the uploading of X-ray images to the TB diagnostic tool by nurses^15^.

Fan et al.^23^ reported that self-diagnosis chatbots were used mostly by younger patients. Although a majority of user feedback for the ‘Doctor Bot’ chatbot was positive, some chatbot users also perceived the provided information to be insufficient, overwhelming, or difficult to understand^23^.

Garzon-Chavez et al.^18^ reported a rather successful incorporation of the chest CT AI-assisted triage tool into the hospital’s COVID-19 triage process which required cases identified as high COVID-19 risk arriving at the emergency room to first undergo CT-based screening. Later during the pandemic, once Reverse Transcription Polymerase Chain Reaction (RT-PCR) tests became more readily available, the AI-assisted chest CT scans remained the dominant form of triage due to its speed despite lower accuracy.

#### Trust in AI systems

User-friendliness is linked to another critical point when introducing AI systems in healthcare: end-user trust in the technologies. Two of our included studies discussed user trust in AI technologies.

Physicians interviewed in Wang et al.^22^ expressed distrust in clinical decision support systems, as the basis on which diagnostic or therapeutic decision-making occurred was not sufficiently transparent. Similarly, Fan et al.^23^ reported that diagnoses produced by the AI self-diagnosis chatbot were perceived as inaccurate by some users.

Wang et al.^24^ further pointed out problematic behaviour by their social support chatbot, whose identity was hidden from end-users on an online social support forum. In one case, in comforting a user who recently had a child, the AI mimicked a human response implying it had the same experience with its own baby. Given the chatbot’s identity was hidden, this raised questions about how AIs should be trained in order to avoid responding inappropriately to user posts^24^.

#### Cost-savings and improvements in health outcomes

Only MacPherson et al. conducted a cost-effectiveness study of their AI tool. Compared to usual care, the AI ‘CAD4TB’ TB-screening tool improved patients’ quality-adjusted life-years (QALYs) by reducing the average time to receive treatment. However, the cost per QALY was measured to be $4,620·47 per QALY gained, which was deemed to be beyond the willingness-to-pay in the Malawian context^19^. Wang et al.^24^ found that an AI chatbot performed comparably with humans in promoting positive responses from online forum users seeking emotional support.

#### Local adequacy of AI

Local adequacy of AI tools was a common theme in our studies, with three studies discussing challenges with applying AI tools to new lower-resource contexts.

Zhou et al.^20^ suggested the US-based training of IBM Watson for Oncology using US medical literature has led to inappropriate treatment suggestions in the Chinese context. Ugarte-Gil et al.^15^ reported unexpected complications with the implementation of their TB diagnostic tool, with their implementation sites having less internet connectivity, X-ray viewer capacity and mobile technology proficiency among health care workers than they had expected, which reduced the effectiveness of the AI tool. Wang et al.^22^ reported the AI clinical decision support tool had not well accounted for rural primary-care physician workflows in its design, and its usefulness could have been improved as a triage assistant rather than a physician assistant.

## Discussion

The literature on AI applications for healthcare in LMICs has been steadily growing in recent years and is so far largely dominated by studies and reports from China and India^26^. Despite the substantial improvements in the technical capabilities of AI in different branches of medicine, such as ophthalmology and radiology^27,28^, many studies were not included in this review because they were proof of concepts and did not describe AI implementations in real-world, low-resource settings, limiting our understanding of the true performance and benefits of AIs^29^. This research is critical to understand both the adaptation to and potential performance of AI tools to medical and other health-related fields in settings where this technology has so far not yet played a strong role^30^. However, we found that researchers are actively addressing this knowledge gap. We came across a rather large number of LMIC-based publications of research protocols related to planned or on-going AI evaluations, as well as studies published since the time we performed our literature search that would have met our inclusion criteria. For instance, recent ophthalmology studies from Thailand and Rwanda have demonstrated the potential of AI-assisted diabetic retinopathy screening in LMICs while also flagging issues similar to those of our included studies, such as the challenge of integrating AI systems into existing workflows and infrastructure^31,32^. The private sector is also highly active in developing AI tools for healthcare, as our grey literature search revealed (see Table 5). None of the AI tools described in the grey literature provide concrete evidence to show that they improve health outcomes, or reduce costs associated with healthcare, although one can assume that some tools are hugely beneficial, such as automated drone deliveries of medical supplies in rural Rwanda^33^. Increased efforts to provide prospective evaluations of such tools would be beneficial for the wider healthcare community by offering lessons in which AI tools can improve health outcomes and/or reduce costs in particular contexts, and what may be required for said AI tools to be successful in their implementation.

**Table 5:** Characteristics of sampled grey literature.

Name of AI tool	Description	Country	Main user group	AI use category	AI type employed
Disease outbreak intelligence platform^65^	Disease outbreak prediction and real-time disease risk assessment for COVID-19	ASEAN countries^66^	Public health practitioners	Population health	Natural language processing; machine learning
Medical robot assistants^67^	Service delivery robots in hospitals to improve patient care	China	Non-physician healthcare workers	Healthcare delivery	Robotics
CT and X-ray diagnostics^68,69^	Computer assisted diagnostic (CAD) radiology tool for COVID-19 and other conditions to aid physicians	China	Physicians	Clinical decision support	Computer vision using deep learning
Close contact catcher^70^	Population surveillance identifying close contact between individuals	China	Public health practitioners	Population health	Computer vision using deep learning
Deep learning - fractional flow reserve derived from coronary CT angiography^71^	Automated non-invasive physiological functional assessment of coronary arteries using coronary CT angiograms as an alternative to invasive coronary angiography	China	Physicians	Clinical decision support	Computer vision using deep learning
Diabetes risk prediction tool^72^	Predictive tool for individual users to identify their risk of diabetes, and to promote early diagnosis	China	Individuals/patients	Population health	Machine learning
Robotic COVID-19 case monitoring^73^	Automated screening calls and follow-up calls, performed by voice robots, in order to reduce call centre workloads	China	Non-physician healthcare workers	Frontline health worker virtual assistant	Robotics
Intelligent triage^74^	Platform for patients to consult with ‘AI Doctor’ in order to facilitate access to relevant medical information and possible diagnoses, and to find a suitable doctor	China	Individuals/patients	Patient virtual assistant	Natural language processing; machine learning
Intelligent hospitals^75^	Integration of multiple AI services, including speech input medical records, CAD systems, and AI-driven follow up	China	Physicians; non-physician healthcare workers	Frontline health worker virtual assistant; clinical decision support	Natural language processing; computer vision using deep learning; machine learning; expert systems
Autonomous drone delivery^33,76–78^	Drone delivery of medical supplies and samples to hospitals	China, Dominican Republic, Rwanda, Madagascar, Malawi, Senegal	Healthcare providers	Healthcare delivery	Robotics
Diabetic retinopathy screening^79,80^	Computer Assisted Diagnostic tool diagnosing diabetic retinopathy using hospital retinal imaging to ease physician workloads	China, India	Physicians; non-physician healthcare workers	Clinical decision support	Computer vision using deep learning
RAD-AID AI radiology^81^	Capacity building and implementation of CAD radiology tools to ease workloads of radiologists in low-resource settings	Multiple Latin American, Asian and African Countries	Physicians; non-physician healthcare workers	Clinical decision support	Computer vision using deep learning
Automated whole slide imaging and histological diagnostics^82^	Automated whole slide imaging using conventional microscopes and smartphone, and AI histology diagnostic assistant, for diagnostics in low-resource settings	Mexico, Tanzania, India	Physicians; non-physician healthcare workers	Frontline health worker virtual assistant; clinical decision support	Computer vision using deep learning
Automated malaria diagnostics^83^	Web-based platform for diagnosing malaria with thick blood smear images to strengthen laboratories	Uganda	Physicians; non-physician healthcare workers	Frontline health worker virtual assistant	Computer vision using deep learning
Health chatbots^84–87^	App and web-based chatbots automating triage and self-directed care for patients	Brazil, China, India, Tanzania	Individuals/patients	Patient virtual assistant	Natural language processing; machine learning
Patient retention in HIV care^88,89^	Predictive tool for healthcare providers to identify HIV patients at risk of being lost to follow up, promoting proactive intervention	South Africa	Healthcare providers	Population health	Machine learning
Tailored healthcare worker training^90^	Identifying recurrent errors by health workers and proposing AI tailored training modules via digital platform	Burkina Faso	Non-physician healthcare workers	Personnel management	Machine learning
Perinatal asphyxia computer aided diagnostics^91^	CAD tool used by healthcare workers to detect early signs of perinatal asphyxia using recordings of newborn cry sounds	Nigeria	Non-physician healthcare workers	Frontline health worker virtual assistant	Computer audition using deep learning

The performance of AI applications in healthcare settings varies greatly, as was also observed in previous reviews of AI applications in medical imaging when compared to clinicians^34,35^. This is similar to included studies in this review that found inconsistencies in diagnostic sensitivity and specificity between AI tools and physician assessments^17,18,20,22^. Also, we were unable to identify many studies performing prospective feasibility testing or trials of AI tools in real-world settings in order to test their performance^34,35^. The reported performance of AI tools tested on retrospective datasets should be treated with caution, as a tool’s accuracy likely diminishes when applied to new data from different contexts^18,35^. Further studies of the performance of AI tools applied in healthcare settings are required to take into account data and concept drift^36^. Based on our review, existing evidence is also limited by inconsistent levels of reported transparency with respect to AI implementation and performance. For instance, there seems to be no systematic approach to report on the use of the type of datasets used for AI training, testing and validation, the underlying training algorithms, and key AI outputs that would allow a more direct comparison of AI performance, as well as to identify potential causes of poor performance^34^.

The underlying dataset is a key element of training the AI tool and its performance. Data from included research suggested that AI systems were trained on data collected outside of the implementation context^17,20,22^. However, AI-models trained on high-income country data may introduce bias into AI outputs, leading to poor performance or, worse, wrong results - which is harmful in a health-context and also harmful in establishing AI in healthcare because trust may be broken. Given that data is dynamic and may change its statistical features over time (data and concept drift), it is critical that AI models receive context-specific and updated data on a frequent basis; otherwise, AI models’ performance may worsen over time. This could lead to a downward spiral, as poor performance is likely to lead to poor acceptance of HCWs and a loss of trust in AI-based systems. While middle-income countries, like China and South Africa, have substantial collections of data pertaining to both the health system and health service delivery at the national and sub-national levels, the selection of training data is more limited in many low-income countries^11^. On the other hand, available context-specific data sets might be underused, untapped, or deemed too limited or inadequate, as the contained information is too asymmetric, asynchronous or varied in type, and spread across locations to facilitate reliable AI training^11^. There are no clear estimates on the amount of training data needed in designing an AI project. To better leverage small datasets in the context of LMICs, additional modelling techniques and simple classifiers should be considered, like the Naive Bayes algorithm, which allows a sufficiently strong learning process if applied to small datasets^37^. While public health institutions, donor-funded programmes and the business sector all generate large volumes of data, such data is often inaccessible to researchers and AI implementers^38^. Data collection and storage is too fragmented, or only intended for very specific purposes, such as programme reporting, policy development, strategic planning and advocacy^39^. Furthermore, some LMICs still face challenges in digitization of routinely collected data, as well as limited digital literacy with respect to data collection and management^38^. Ongoing efforts to harmonise fragmentations in health information systems that foster accurate, reliable, timely, interoperable datasets will be crucial in advancing AI technologies^38^. Routine data collecting platforms, such as OpenMRS or DHIS2, are well-established in low- and middle-income countries, and other initiatives, such as Health and Demographic Surveillance Systems^40^, provide enormous and standardised population health datasets encompassing decades. Yet, data ownership and data sharing rules can still pose barriers to accessing this data for research and commercial purposes. The Nairobi data sharing guidelines of 2014, as well as the Global Digital Health Index, are both first steps toward finding solutions to this topic. In order to develop datasets that may be used for AI, privacy regulations, data access and ownership agreements, and other essential challenges must be overcome. Public health agencies can play an important role in encouraging data sharing and providing public access to health data – both internal and private-sector generated health data – while also developing the governance mechanisms required to protect individual privacy.

Usability and integration of digital health tools, including AI tools, remain a challenge in high- and low-resource settings alike. Coiera^41^ and Cabitza et al.^42^ identified some of the complex challenges of the “last mile of implementation” that cause a poor translation of statistically high-performing AI into real-world applications. Especially in low-resource settings, the effectiveness of AI tools depends on how well these technologies can be utilised or integrated by end-users within an existing infrastructure^43^. In order to perform well in a real-world setting, AI tools should complement existing organisational networks of people, processes and technologies^41,42^. Inadequate design of user-interfaces can further limit the positive impact of an AI tool on clinical applicability, irrespective of diagnostic accuracy^42^. Complex or confusing user interfaces can lead to frustration among end-users or limited successful tool application, negatively impacting the uptake of technologies by front-line health workers or patients in low-resource settings^15,22,44^. Successful introduction of novel digital tools in low-resource settings therefore needs to account for and increase the basic capacity of HCWs to adopt technologically complex tools^44^. In some of the studies included in our review, AI integration was limited due to incompatibility with existing electronic health record systems, which in turn limited its performance as decisions could not be fully supported by relevant health record data. Another barrier to successful AI implementation includes the often unstable internet connectivity in some low-resource areas, since poor or intermittent internet access disrupts the use of cloud-based tools needed to upload key data elements, such as radiology images^44^.

Trust and acceptance of users is a critical feature of AI for global health and healthcare in general. Trust in AI applications has been found to be stronger if a technology and algorithms are understandable and assist users toward their goals^45^. A majority of reviewed studies still relied on a ‘black box’ approach, which leaves it unclear how the algorithms used eventually arrive at results. Furthermore, only half of studies provided a transparent description of their AI methodologies. Healthcare AI should be transparent about the source of data, qualify AI-based suggestions, and be explainable when they are used by clinicians and patients to make decisions^46^. Otherwise, it could negatively affect the trust foundations and increase the likelihood of rejection of the healthcare AI technology. Of course, patient data security is an essential aspect, particularly as cyberattacks get more sophisticated^47^. The adoption of approaches and structures similar to those regulating the pharma industry and the production of medicines might therefore be a feasible path forward for AI in healthcare. Likewise, AI healthcare applications may need to go through a similar process of preclinical research, clinical research, authority evaluation and post-market safety monitoring. It is also necessary to investigate future revisions of medical curricula to incorporate elements strengthening future HCW’s digital literacy and knowledge which may increase trust and effective usage of technologies, such as AI-based systems. Currently, users often have not received sufficient training and feel overwhelmed. Therefore, digital systems are often regarded as additional burdens. Another approach that appears to build user trust and hence potentially boost technology acceptance is the slow introduction of innovations, which “allows for incremental social learning”^45^. In general, technology acceptance is a complex process^48^. Other factors, such as a thorough understanding of the users’ benefits in contrast to other available technologies and pathways, undoubtedly play an essential part in lessening innovation resistance. It seems beneficial to proactively communicate from the start of the development process^45^. Overall, trust is a complex and delicate component and should be a key priority particularly at the start of the wider implementation of AI-based healthcare applications. Otherwise users, both patients and health care workers, may reject the technologies and impede further progress.

Affordability is an important characteristic of AI tools in a LMIC context. Even if the technologies are efficacious, this benefit cannot be realised if they are more expensive than legacy approaches to which HCWs are familiar. Our review and the wider literature suggest there is a dearth of evidence on the improvements in health outcomes and cost-savings associated with the implementation of AI tools in any context^49^, and of eHealth tools more generally^50^. We hypothesise that this finding reflects the maturity of AI healthcare research, since cost-effectiveness analyses necessarily occur later in the AI tool development and implementation timeline. To evaluate whether AI tools are affordable in LMICs, there is a need for more cost-effectiveness analysis studies.

A number of local challenges were reported in the studies included in this review as well as the wider literature. AI-based systems were not sufficiently integrated in existing workflows and infrastructure; healthcare centres in LMICs are subject to system outages caused by power or internet connectivity disruptions^15,32,51^, and, as a result of donor-funded agendas in LMICs, there is intermittent advancement that is susceptible to trends or “fashions”^38^, eroding faith in these systems further due to their lack of utility and continuity. Additionally, there seems to be a concern among HCWs in LMICs that AI may eventually take over their jobs, impeding its further adoption^52^ .AI applications in healthcare require a holistic systems approach to implementation. Consideration of the multiple interacting facilitators and barriers to AI implementation in real-world settings is required, in addition to the technical performance AI system in addressing a specific health problem, in order to have maximal impact on human health. Future implementations may also want to consider ‘effective coverage’ - the need, use and quality of health intervention - as a performance metric^53^. Further studies are required in order to address contextual challenges, such as trust and HCW job insecurity, data insecurity and sustainability, in order to inform future AI implementations in healthcare in LMICs.

Although we attempted to perform a broad search of studies of AI deployed in healthcare in LMICs when performing our database search, we may have missed important papers that would have met our inclusion criteria. We mitigated the risk of this by also performing Google Scholar search with broad search terms, as well as exploring grey literature extensively, looking at papers cited in multiple reviews of AI in healthcare and research presented at various AI and healthcare conferences. Only articles published in English were included. This is a limitation of the review since China is an area with a highly active AI research field. However, there are research articles published in English, produced in China, that we were able to include in the review. Articles also had to have been peer-reviewed, which notably excluded a small number of recently published manuscripts on https://arxiv.org/. We concentrated exclusively on completed studies, which may have resulted in a significant reduction in the number of papers, leaving out ongoing research activity that may have been communicated via other channels. The field of AI research is rapidly evolving, therefore our review has also excluded relevant new research that has been published between the time of our database search and the publishing of this paper.

This systematic review has identified ten articles where a wide variety of AI technologies that have been implemented in varying healthcare settings across seven LMICs. AI has a demonstrated potential in triage, diagnostics and treatment planning settings. However, many challenges and barriers to successful implementation exist. Greater transparency and availability of algorithms and datasets used to train AIs could allow for a great understanding of why particular tools perform well or poorly. Further studies of AI use-cases in healthcare settings are required along a number of avenues, including: prospective studies that demonstrate real-world reliability and cost-effectiveness of AI tools, analyses of end-user perspectives of AI usability and trust in AI technologies, and how to effectively integrate AI systems into existing healthcare infrastructure.

## Methods

To identify and map all relevant AI studies in LMICs that addressed our research questions, we considered a systematic scoping review as the most suitable methodology for our evidence review^54^. We followed five iterative stages as described by Arksey and O’Malley and systematically reviewed identified literature in line with published scoping review guidelines^55–57^. We report our findings in accordance with the Preferred Reporting Items for System Reviews and Meta-Analyses Extension for Scoping Reviews (PRISMA-ScR)^58^.

### Databases searched

Our literature search included five electronic databases: Scopus, EMBASE, MEDLINE, Global Health and APA PsycInfo. A search strategy for each database was developed to identify relevant studies (see Supplementary Table 1 for search terms used). We further expanded our search to include grey literature via Google Scholar^59^. We also conducted a handsearch of journals and conference papers discussing AI applications in global health.

Overall, we included only peer-reviewed literature. Since the field of AI in healthcare is a rapidly evolving field, numerous publications were available ahead of print. In these instances, we only included pre-prints that had already undergone at least initial peer-review. We also reviewed papers presented at AI conferences, as it is common in the field of AI that publications are made available at key conferences which also peer-review submissions.

### Search criteria

We applied a variety of search terms consisting of concepts related to AI, healthcare, and LMICs to identify a broad range of peer-reviewed, original records on AI, health and healthcare in LMICs. Our literature search included records published between 1st January 2009 and 30th September 2021. We limited our search to literature published after 2009, as this year marks the point at which graphic processing units (GPUs) were repurposed for AI applications, thus providing a substantial boost in the speed at which AI models could be trained and implemented^60^. LMICs were defined based on the World Bank Group Classification of Economies as of January 2021^61^. We only included records describing original studies. Records without full-text and articles such as commentaries, letters, policy briefs and study protocols were excluded. Our search further included records that described a quantitative and/or qualitative evaluation of an implemented AI application related to healthcare. Hence, studies merely describing theoretical AI approaches, such as machine learning methods in a non-specific or non-LMIC context without defining a real-world application of AI in a LMIC health context, were not considered.

### Study screening and selection

Records identified by the above database searches were entered into the Covidence Systematic Review Software for further title and abstract review^62^. Inclusion and exclusion criteria were identified following the PICOS (population, intervention, comparison, outcome, study design) framework (see Table 6 for details)^63^. Three reviewers (T.C.H., R.S. and M.A.) screened titles and abstracts independently to select those articles fully meeting set inclusion criteria related to the application of AI in healthcare in an LMIC. Discrepancies in reviewer ratings were discussed and decided within the entire research team (T.C.H., R.S., M.A., St.B. and S.B.). Once relevant articles had been identified, the reviewers (T.C.H., R.S. and M.A.) screened all full texts to exclude those articles which did not meet inclusion based on full-text review.

**Table 6:** Inclusion and exclusion criteria based on the PICOS (population, intervention, comparison, outcome and study design) framework.

	Inclusion	Exclusion
Population	Health care workers and/or patients, given AI implemented in a country defined as a low- and middle-income country	Non-health-related sample Health care workers, given AI implementation in high-income or unspecified country
Intervention	AI implemented in global health context	AI not implemented, only theoretically described Focus on model testing, no real-world application
Comparison	Comparison of AI intervention to standard methods Qualitative evaluation of sample population to AI intervention	No form of comparison conducted
Outcome	Evaluation of AI in global health context	AI used as a secondary tool to analyse another outcome
Study design	Any primary research, qualitative or quantitative Full text available Peer-reviewed	Secondary/synthesis research Only abstract available Commentaries Letters, letters to editor Policy briefs Study protocols
Language	English	Non-English full-text
Time frame	Published after 1st January 2009	Published until 31st December 2008

### Data extraction and synthesis

We used a data extraction form to chart characteristics and map key findings from the final set of articles (see Supplementary Fig. 1). Key AI characteristics included aspects such as the application field and context, dataset sources and algorithms used. Additionally, we mapped the specific use of each AI application as an assistant for either patients, health workers, or physicians^64^. We extracted descriptive and methodological characteristics of each reviewed study. Content mapping focused on extracting and comparing as well as pertinent outcomes and reported lessons learned.

### Reporting summary

Further information on research design is available in the Nature Research Reporting Summary linked to this article.

## Supplementary information

Supplementary Material

NPJ Reporting Summary

## Data Availability

All data generated and analysed during this study are included in the article and its supplementary information files.
