# Supplementary material for: Artificial intelligence for strengthening healthcare systems in low- and middle-income countries: a systematic scoping review
Source: NPJ Digit Med. 2022 Oct 28;5:162. doi: 10.1038/s41746-022-00700-y (PMC9614192; doi:10.1038/s41746-022-00700-y)
Supplement: Supplementary file 1 — Supplementary Material [file 41746_2022_700_MOESM1_ESM.pdf]

Supplementary File

Contents **1**

Supplementary Table 1 - Database Search Terms **1**

Supplementary Note 1 - Google Scholar Search Terms **1**

Supplementary Figure 1 - Data Extraction Form **3**

Supplementary Note 2 - References for studies included in the review in alphabetical order **6**

Supplementary Table 2 - AI algorithm types and datasets used in included studies **7**

Supplementary Discussion - AI algorithms and training datasets of included studies **7**

Supplementary References **9**

Supplementary Table 1 - Database Search Terms

**Search Term Category**	**Searches**
AI Terms	"Artificial intelligence" OR "AI" OR "abductive logic programming" OR "ALP" OR "ambient intelligent" OR "AmI" OR "artificial neural network" OR "ANN" OR "automated planning" OR "automated scheduling" OR "automated reasoning" or "automatic computing" OR "autonomous robot" OR "behavior tree" OR "belief desire intention model" OR "big data" OR "brain technology" OR "capsule neural network" OR "capsule network" OR "CapsNet" OR "cased based reasoning" OR "CBR" OR "cloud robotics" OR "cluster analysis" OR "cognitive architecture" OR "cognitive computing" OR "committee machine" OR "commonsense reasoning" OR "computation creativity" OR "computational cybernetics" OR "computational intelligence" OR "computational linguistics" OR "computational neuroscience" OR "computer automated design" OR "CAD" OR "computer audition" OR "computer vision" OR "convolutional neural network" OR "CNN" OR "data mining" OR "decision support system" OR "DSS" OR "decision tree" OR "deep learning" OR "distributed artificial intelligence" OR "error driven learning" OR "ensemble averaging" OR "evolutionary algorithm" OR "expert system" OR "fuzzy logic" OR "fuzzy control system" OR "machine vision" OR "markov decision process" OR "machine learning" OR "ML" OR "machine listening" OR "machine perception" OR "naive bayes classif*" OR "natural language processing" OR "NLP" OR "random forest" OR "recurrent neural network" OR "RNN" OR "reinforcement learning" OR "RL" OR "supervised learning" OR "support vector machine" OR "SVM" OR "swarm intelligence" OR "unsupervised learning"
Health system terms	"health workforce" OR "surveillance system" OR "health governance" OR "health system strengthening" OR "resilient health system" OR "health system" OR "service delivery" OR "health workforce" OR "financing" OR "leadership" OR "governance" OR "health information system*" OR "access to medicine"
LMIC terms	“low income econom*" OR "lower income econom*" OR "low income countr*" OR "lower income countr*" OR "afghanistan" OR "burkina faso" OR "burundi" OR "central african republic" OR "chad" OR "congo" OR "democratic republic congo" OR "eritria" OR "ethiopia" OR "gambia" OR "guinea" OR "guinea bissau" OR "haiti" OR "north korea" OR "liberia" OR "madagascar" OR "malawi" OR "mali" OR "mozambique" OR "niger" OR "rwanda" OR "sierra leone" OR "somalia" OR "south sudan" OR "sudan" OR "syria" OR "syrian arab republic" OR "tajikistan" OR "togo" OR "uganda" OR "yemen" OR "lower middle income countr*" OR "lower middle income econom*" OR "algeria" OR "bangladesh" OR "benin" OR "bhutan" OR "bolivia" OR "cabo verde" OR "cape verde" OR "cambodia" OR "cameroon" OR "comoros" OR "republic of congo" OR "côte d'Ivoire" OR "cote d'ivoire" OR "ivory coast" OR "dijbouti" OR "egypt" OR "el salvador" OR "eswatini" OR "ghana" OR "honduras" OR "india" OR

	"kenya" OR "kiribati" OR "kyrgyz republic" OR "kyrgyzstan" OR "lao pdr" OR "laos" OR "lesotho" OR "mauritania" OR "micronesia" OR "moldova" OR "mongolia" OR "morocco" OR "myanmar" OR "nepal" OR "nicaragua" OR "nigeria" OR "pakistan" OR "papua new guinea" OR "philipines" OR "são tomé and principe" OR "saint thomas and prince" OR "senegal" OR "solomon islands" OR "sri lanka" OR "tanzania" OR "timor leste" OR "east timor" OR " tunisia" OR "ukraine" OR "uzbekistan" OR "vanuatu" OR "vietnam" OR "west bank" OR "gaza" OR "palastine" OR "zambia" OR "zimbabwe" OR "middle income econom*" OR "middle income countr*" OR "albania" OR "american samoa" OR "argentina" OR "armenia" OR "azerbaijan" OR "belarus" OR "belize" OR "bosnia" OR "herzegovina" OR "botswana" OR "brazil" OR "brasil" OR "bulgaria" OR "china" OR "colombia" OR "costa rica" OR "cuba" OR "dominica" OR "dominican republic" OR "equatorial guinea" OR "ecuador" OR "fiji" OR "gabon" OR "georgia" OR "grenada" OR "guatemala" OR "guyana" OR "indonesia" OR "iran" OR "iraq" OR "jamaica" OR "jordan" OR "kazakhstan" OR "kosovo" OR "lebanon" OR "libya" OR "malaysia" OR "maldives" OR "marshall islands" OR "mexico" OR "montenegro" OR "namibia" OR "north macedonia" OR "macedonia" OR "paraguay" OR "peru" OR "russia" OR "samoa" OR "serbia" OR "south africa" OR "st lucia" OR "saint lucia" OR "st vincent and the grenadines" OR "saint vincent" OR "grenadines" OR "suriname" OR "thailand" OR "tonga" OR "turkey" OR "turkmenistan" OR "tuvalu" OR "venezuala" OR "sub saharan africa" OR "ssa" OR "east asia" OR "south asia" OR "middle east" OR "east europe" OR "central asia" OR "latin america" OR "caribbean" OR "lower income population?" OR "underserved countr*" OR "underserved nation?" OR "underserved population?" OR "underserved world" OR "under served countr*" OR "under served nation?" OR "under served population?" OR "under served world" OR "deprived countr*" OR "deprived nation?" OR "deprived population?" OR "deprived world" OR "poor countr*" OR "poor nation?" OR "poor population?" OR "poor world" OR "poorer countr*" OR "poorer nation?" OR "poorer population?" OR "poorer world" OR "developing econom*" OR "lmic" OR "lmics"
Overall Search	AI terms AND Health system terms AND LMIC terms

Using the Ovid platform, 'all fields' (.af) were searched for AI terms and health system terms, and just titles (.ti) were searched for LMIC terms. Using the SCOPUS platform, only titles (.ti) were searched for all search terms.

Supplementary Note 1 - Google Scholar Search Terms

"low- middle- income countries" OR "LMICs" AND "artificial intelligence" AND "health"

Supplementary Figure 1 - Data Extraction Form

DATA EXTRACTION

General information

Title

Title of paper / abstract / report that data are extracted from

Lead author contact details

Country in which the study conducted

Notes

Characteristics of included studies

Methods

Aim of study

Study design

- (●) Randomised controlled trial
- ( ) Non-randomised experimental study
- ( ) Cohort study
- ( ) Cross sectional study
- ( ) Case control study
- ( ) Systematic review
- ( ) Qualitative research
- ( ) Prevalence study
- ( ) Case series
- ( ) Case report
- ( ) Diagnostic test accuracy study
- ( ) Clinical prediction rule
- ( ) Economic evaluation
- ( ) Text and opinion
- ( ) Other

Clear above selection

Start date

End date

Study Funding Sources

Possible conflicts of interest for study authors

Participants

Population description

Inclusion criteria

Exclusion criteria

Method of recruitment of participants

- ○ Phone
- ○ Mail
- ○ Clinic patients
- ○ Voluntary
- ○ Other

Clear above selection

Total number of participants

Information About AI Used

Healthcare Application

AI Algorithms Used

Types of algorithms used

AI Category

Category of AI - e.g. computer vision, process automation, robotics

Performance of AI

Strengths

Weaknesses

Perceptions (from Health Care Workers) in relation to

Clinical application/user-friendliness

Patient outcomes

Costs, additional resources needed (staff, equipment, etc.)

Data Sources and Management

How did they generate data?

Who generated what kind of data? What are the processes involved?

What kind of data goes into the AI?

What is the quality of said data?

How is the output of the AI digested?

I.e. how is the AI output processes and used/ interpreted?

Evaluation of AI Outputs

What the key findings of the paper?

What are the lessons learned?

Any further steps planned?

Supplementary Note 2 - References for studies included in the review in alphabetical order

Fan, X. *et al.* Utilization of Self-Diagnosis Health Chatbots in Real-World Settings: Case Study. *J. Med. Internet Res.* **23**, e19928 (2021).

Ganju, A., Satyan, S., Tanna, V. & Menezes, S. R. AI for Improving Children’s Health: A Community Case Study. *Front. Artif. Intell.* **3**, 544972 (2021).

Garzon-Chavez, D. *et al.* Adapting for the COVID-19 pandemic in Ecuador, a characterization of hospital strategies and patients. *PLoS ONE* **16**, e0251295 (2021).

Kisling, K. *et al.* Fully Automatic Treatment Planning for External-Beam Radiation Therapy of Locally Advanced Cervical Cancer: A Tool for Low-Resource Clinics. *J. Glob. Oncol.* 1–9 (2019) doi:10.1200/JGO.18.00107.

Love, S. M. *et al.* Palpable Breast Lump Triage by Minimally Trained Operators in Mexico Using Computer-Assisted Diagnosis and Low-Cost Ultrasound. *J. Glob. Oncol.* 1–9 (2018) doi:10.1200/JGO.17.00222.

MacPherson, P. *et al.* Computer-aided X-ray screening for tuberculosis and HIV testing among adults with cough in Malawi (the PROSPECT study): A randomised trial and cost-effectiveness analysis. *PLOS Med.* **18**, e1003752 (2021).

Ugarte-Gil, C. *et al.* Implementing a socio-technical system for computer-aided tuberculosis diagnosis in Peru: A field trial among health professionals in resource-constraint settings. *Health Informatics J.* **26**, 2762–2775 (2020).

Wang, D. *et al.* “Brilliant AI Doctor” in Rural Clinics: Challenges in AI-Powered Clinical Decision Support System Deployment. in *Proceedings of the 2021 CHI Conference on Human Factors in Computing Systems* 1–18 (ACM, 2021). doi:10.1145/3411764.3445432.

Wang, L. *et al.* CASS: Towards Building a Social-Support Chatbot for Online Health Community. *Proc. ACM Hum.-Comput. Interact.* **5**, 9:1-9:31 (2021).

Zhou, N. *et al.* Concordance Study Between IBM Watson for Oncology and Clinical Practice for Patients with Cancer in China. *The Oncologist* **24**, 812–819 (2019).

Supplementary Table 2 - AI algorithm types and datasets used in included studies

Characteristics	No. of studies (%)
**AI algorithms/approaches used**	
Machine learning	10 (100%)
Deep learning	7 (70%)
Natural language processing	4 (40%)
Automatic image segmentation	2 (20%)
Knowledge graphs	2 (20%)
**Datasets used**	
Dataset referenced	5 (50%)
Dataset sourced from local context	3 (30%)
Dataset not referenced	5 (50%)

Supplementary Discussion - AI algorithms and training datasets of included studies

AI algorithms studied

Supplementary Table 2 provides a summary of the types of algorithms used in the included studies. Machine learning was used by all the AI tools examined by studies included in this review, however, the underlying algorithms in use varied depending on the AI application. In the study by Ganju et al. (2021), random forest modelling was used to predict user churn on an mHealth app. In many studies (60%) deep-learning methods were applied to both computer vision and natural language processing.[1–6] For the computer-aided detection for tuberculosis (CAD4TB) and the Radiation Planning Assistant,[7,8] the authors described non deep-learning automatic segmentation techniques at the time of study, although current versions of the tools now also apply deep-learning. Three papers (30%) reporting on the use of commercial tools provided only superficial detail on the underlying algorithms.[3–5] Convolutional Neural Networks (CNNs) were examined by three papers (30%), such as the use of GoogLeNet Inception v3 CNN architecture,[9] and a CNN architecture for text classification.[6] Wang et al. (2021b) reported the use of OpenNMT, an open-source tool for neural machine

translation that uses Recurrent Neural Networks (RNNs) for text generation.[10] The use of Natural Language Processing (NLP) techniques was reported for both the chatbots and the clinical decision support systems (n=4, 40%).[4–6,11] NLP, in addition to knowledge graphs (n=2, 20%), was reported as a means of accessing relevant clinical information.[4,5] Of these papers, only Wang et al. (2021b) described these applied NLP techniques used in detail.[6]

Datasets used

Five studies provide reference to the datasets used.[1–3,6,12] In the study by Love et al. (2018), the CNN model was trained using breast ultrasound images from multiple sources in the United States, before testing the model on images collected in Mexico.[1] In outlining the development of its TB triage tool, Ugarte-Gil et al. (2020) references their previously published paper, describing the use of ImageNet as a pre-training dataset, and a dataset of 4,701 chest X-ray images from Peru which they used to tune the model.[2,13] Ganju et al. (2021) and Wang et al. (2021b) reference the mHealth app and online chatboard used to train their models, as well as the dimensions used.[6,12] Garzon-Chavez et al. (2021) provided limited information regarding their training set of the commercially available COVID-19 diagnostic tool, but mentioned that this included 4,000+ chest CTs from confirmed COVID-19 patients in China.[3,14]

The studies by Zhou et al. (2019), Fan et al. (2021), Wang et al. (2021a), and MacPherson et al. (2021) also used commercially available products (i.e., the IBM Watson for Oncology, DoctorBot, the Brilliant Doctor, and Delft's CAD4TB) but provide limited information on their respective datasets, other than that the IBM Watson for Oncology was trained using oncology literature primarily sourced from the US context, and the tools developed for the DoctorBot and Brilliant Doctor used datasets from the Chinese context.[4,5,7,11]

Supplementary References

1 Love, S. M. *et al.* Palpable Breast Lump Triage by Minimally Trained Operators in Mexico Using Computer-Assisted Diagnosis and Low-Cost Ultrasound. *J. Glob. Oncol.* 1–9 (2018) doi:10.1200/JGO.17.00222.

2 Ugarte-Gil C, Icochea M, Llontop Otero JC, *et al.* Implementing a socio-technical system for computer-aided tuberculosis diagnosis in Peru: A field trial among health professionals in resource-constraint settings. *Health Informatics J* 2020; **26**: 2762–75.

3 Garzon-Chavez, D. *et al.* Adapting for the COVID-19 pandemic in Ecuador, a characterization of hospital strategies and patients. *PLoS ONE* **16**, e0251295 (2021).

4 Fan, X. *et al.* Utilization of Self-Diagnosis Health Chatbots in Real-World Settings: Case Study. *J. Med. Internet Res.* **23**, e19928 (2021).

5 Wang, D. *et al.* "Brilliant AI Doctor" in Rural Clinics: Challenges in AI-Powered Clinical Decision Support System Deployment. in *Proceedings of the 2021 CHI Conference on Human Factors in Computing Systems* 1–18 (ACM, 2021). doi:10.1145/3411764.3445432.

6 Wang, L. *et al.* CASS: Towards Building a Social-Support Chatbot for Online Health Community. *Proc. ACM Hum.-Comput. Interact.* **5**, 9:1-9:31 (2021).

7 MacPherson, P. *et al.* Computer-aided X-ray screening for tuberculosis and HIV testing among adults with cough in Malawi (the PROSPECT study): A randomised trial and cost-effectiveness analysis. *PLOS Med.* **18**, e1003752 (2021).

8 Kisling, K. *et al.* Fully Automatic Treatment Planning for External-Beam Radiation Therapy of Locally Advanced Cervical Cancer: A Tool for Low-Resource Clinics. *J. Glob. Oncol.* 1–9 (2019) doi:10.1200/JGO.18.00107.

9 Szegedy, C. *et al.* Going deeper with convolutions. in *2015 IEEE Conference on Computer Vision and Pattern Recognition (CVPR)* 1–9 (IEEE, 2015). doi:10.1109/CVPR.2015.7298594.

10 Klein, G. *et al.* OpenNMT: Neural Machine Translation Toolkit. *arXiv:1805.11462* (2018).

11 Zhou, N. *et al.* Concordance Study Between IBM Watson for Oncology and Clinical Practice for Patients with Cancer in China. *The Oncologist* **24**, 812–819 (2019).

12 Ganju, A., Satyan, S., Tanna, V. & Menezes, S. R. AI for Improving Children's Health: A Community Case Study. *Front. Artif. Intell.* **3**, 544972 (2021).

13 Alcantara, M. F. *et al.* eRx – A technological advance to speed-up TB diagnostics. *Smart Health* **16**, 100117 (2020).

14 AI-assisted CT Chest Screening for COVID-19-HUAWEI CLOUD Marketplace. https://marketplace.huaweicloud.com/intl/product/00301-207946-0--0.
