# Supplementary material for: Artificial intelligence for strengthening healthcare systems in low- and middle-income countries: a systematic scoping review
Source: NPJ Digit Med. 2022 Oct 28;5:162. doi: 10.1038/s41746-022-00700-y (PMC9614192; doi:10.1038/s41746-022-00700-y)
Supplement: Supplementary file 2 — NPJ Reporting Summary [file 41746_2022_700_MOESM2_ESM.pdf]

nature portfolio

Corresponding author(s): Tadeusz Ciecierski-Holmes

Last updated by author(s): 14/09/2022

Reporting Summary

Nature Portfolio wishes to improve the reproducibility of the work that we publish. This form provides structure for consistency and transparency in reporting. For further information on Nature Portfolio policies, see our Editorial Policies and the Editorial Policy Checklist.

Statistics

For all statistical analyses, confirm that the following items are present in the figure legend, table legend, main text, or Methods section.

n/a	Confirmed	
☐	☒	The exact sample size (n) for each experimental group/condition, given as a discrete number and unit of measurement
☒	☐	A statement on whether measurements were taken from distinct samples or whether the same sample was measured repeatedly
☒	☐	The statistical test(s) used AND whether they are one- or two-sided<br>*Only common tests should be described solely by name; describe more complex techniques in the Methods section.*
☒	☐	A description of all covariates tested
☒	☐	A description of any assumptions or corrections, such as tests of normality and adjustment for multiple comparisons
☒	☐	A full description of the statistical parameters including central tendency (e.g. means) or other basic estimates (e.g. regression coefficient) AND variation (e.g. standard deviation) or associated estimates of uncertainty (e.g. confidence intervals)
☒	☐	For null hypothesis testing, the test statistic (e.g. F, t, r) with confidence intervals, effect sizes, degrees of freedom and P value noted<br>*Give P values as exact values whenever suitable.*
☒	☐	For Bayesian analysis, information on the choice of priors and Markov chain Monte Carlo settings
☒	☐	For hierarchical and complex designs, identification of the appropriate level for tests and full reporting of outcomes
☒	☐	Estimates of effect sizes (e.g. Cohen's d, Pearson's r), indicating how they were calculated

Our web collection on statistics for biologists contains articles on many of the points above.

Software and code

Policy information about availability of computer code

Data collection	The Ovid platform was used to perform our database searches, alongside Google Scholar, using search terms provided in the supplementary material.
Data analysis	The Covidence platform was used to assist with the systematic review process, including data extraction.

For manuscripts utilizing custom algorithms or software that are central to the research but not yet described in published literature, software must be made available to editors and reviewers. We strongly encourage code deposition in a community repository (e.g. GitHub). See the Nature Portfolio guidelines for submitting code & software for further information.

Data

Policy information about availability of data

All manuscripts must include a data availability statement. This statement should provide the following information, where applicable:

- Accession codes, unique identifiers, or web links for publicly available datasets
- A description of any restrictions on data availability
- For clinical datasets or third party data, please ensure that the statement adheres to our policy

All data generated and analysed during this study are included in the article and its supplementary information files.

Human research participants

Policy information about studies involving human research participants and Sex and Gender in Research.

Reporting on sex and gender	Our manuscript makes no explicit mention of sex or gender, and neither sex or gender were a theme in our results or discussion. All references to individuals use gender neutral language.
Population characteristics	Our work reviewed studies involving human populations. We provide some summary information about the populations used in these studies, such as their low- and middle-income country location and how they interact with the AI technologies reviewed in this study.
Recruitment	We did not recruit any human research participants.
Ethics oversight	We did not require ethics approval for this study.

Note that full information on the approval of the study protocol must also be provided in the manuscript.

Field-specific reporting

Please select the one below that is the best fit for your research. If you are not sure, read the appropriate sections before making your selection.

☒ Life sciences ☐ Behavioural & social sciences ☐ Ecological, evolutionary & environmental sciences

For a reference copy of the document with all sections, see nature.com/documents/nr-reporting-summary-flat.pdf

Life sciences study design

All studies must disclose on these points even when the disclosure is negative.

Sample size	Sample size of ten papers relating to AI applications in healthcare in low- and middle-income countries, drawn from a sample of 1126 papers from database and handsearches of the literature.
Data exclusions	We excluded papers from a search databases and handsearches, narrowing down our sample of 1126 papers to 10 based on our inclusion and exclusion criteria stated in the methods section.
Replication	We have presented our search terms in the supplementary material. Repeats of our searches of the databases on the Ovid with the parameters we have laid out in our methods section produce replicable results. Google Scholar searches are not as replicable, but repeating the search with the search terms we used yield similar samples of papers. Handsearching relevant papers is a less replicable practice, and may yield a different sample of papers depending on where researchers search - though this practice only contributed to small minority of our papers.
Randomization	Randominzation is not relevant to our study. During the screening process, two or more researchers had to screen a paper before it could be included or excluded. Each researcher could select which papers to screen from a list.
Blinding	During the screening process, reviewers could not see the decisions made by other researchers until after they had made a decision to include/exclude a study. When there was discordance in inclusion/exclusion decisions, the research team would discuss the paper together in order to decide whether a study should be include or excluded.

Reporting for specific materials, systems and methods

We require information from authors about some types of materials, experimental systems and methods used in many studies. Here, indicate whether each material, system or method listed is relevant to your study. If you are not sure if a list item applies to your research, read the appropriate section before selecting a response.

Materials & experimental systems

n/a	Involved in the study	
☒	☐	Antibodies
☒	☐	Eukaryotic cell lines
☒	☐	Palaeontology and archaeology
☒	☐	Animals and other organisms
☒	☐	Clinical data
☒	☐	Dual use research of concern

Methods

n/a	Involved in the study	
☒	☐	ChIP-seq
☒	☐	Flow cytometry
☒	☐	MRI-based neuroimaging
